# Dynamic Spectrum Access for Internet of Things Service in Cognitive Radio-Enabled LPWANs

**DOI:** 10.3390/s17122818

**Published:** 2017-12-05

**Authors:** Bongkyo Moon

**Affiliations:** Department of Computer Science and Engineering, Dongguk University-Seoul, 30 Pildong-ro 1 gil, Jung-gu, Seoul 04620, Korea; bkmoon@dongguk.edu; Tel.: +82-2-2260-8592

**Keywords:** IoT, cognitive radio, dynamic spectrum access, matrix geometric solution, mean dwell time, carried traffic

## Abstract

In this paper, we focus on a dynamic spectrum access strategy for Internet of Things (IoT) applications in two types of radio systems: cellular networks and cognitive radio-enabled low power wide area networks (CR-LPWANs). The spectrum channel contention between the licensed cellular networks and the unlicensed CR-LPWANs, which work with them, only takes place within the cellular radio spectrum range. Our aim is to maximize the spectrum capacity for the unlicensed users while ensuring that it never interferes with the licensed network. Therefore, in this paper we propose a dynamic spectrum access strategy for CR-LPWANs operating in both licensed and unlicensed bands. The simulation and the numerical analysis by using a matrix geometric approach for the strategy are presented. Finally, we obtain the blocking probability of the licensed users, the mean dwell time of the unlicensed user, and the total carried traffic and combined service quality for the licensed and unlicensed users. The results show that the proposed strategy can maximize the spectrum capacity for the unlicensed users using IoT applications as well as keep the service quality of the licensed users independent of them.

## 1. Introduction

In general, service providers (SPs) for public radio communication need to pay the government the high cost for licensing of radio spectrum bands in the respective country. Once the spectrum bands are assigned, the amount of available spectrum in each band is fixed. In contrast, the industrial, scientific and medical (ISM) radio bands are exceptionally unlicensed radio spectrum although they are served with fixed spectrum allocation as well. Moreover, the unlicensed ISM band is suitable for building Internet of Things (IoT) services with flexibility and low cost without any restrictions on wireless coverage and network topology [1,2]. The traditional wireless sensor networks (WSNs) applications operate most commonly based on the 2.4 GHz unlicensed band under IEEE 802.15.4 standard. However, this band has often been overcrowded due to other wireless technologies such as WPANs (wireless personal area networks), WLANs (wireless local area networks), and WiMAX (worldwide interoperability for microwave access) operating over the same bands [1,3]. Recently, low power wide area network (LPWAN) systems such as LoRa [2,4,5], SigFox [2,5], and Wi-SUN [6], which operate most commonly under 800~900 MHz unlicensed bands, have been deployed commercially for IoT services even though the 3GPP NB-IoT [5,7] system still uses the licensed bands.

Since many licensed spectrum bands are often either underutilized or unutilized, unlicensed users can temporarily use the unused spectrum such as spectrum hole without interfering with licensed users. Cognitive radio (CR) techniques have been used to alleviate the limitations of spectrum in wireless networks. However, cognitive radio has a critical drawback that it must not cause radio spectrum interference to the licensed band. CR can avoid spectrum interference by altering its transmission. The CR can provide a network or a wireless node with the abilities to sense its environment such as spectrum holes, and change its transmitter parameters during communication. Thus, the CR attempts to get the best available spectrum through the interaction with its spectrum environment. That is, one aim of CR is to maximize the spectrum utilization for scare radio resources [8,9,10,11,12,13]. Until now, many researches have been done in order to cope with the coexistence of multiple wireless technologies in unlicensed spectrum bands in the WSNs [14,15,16,17,18,19,20,21].

Similarly, IoT devices can also exploit the potential advantages of cognitive techniques if they are equipped with CR. That is, they can manage the radio spectrum dynamically in the aid of the cognitive radio capabilities. The cognitive radio can be one of the most promising techniques for IoT systems since it improves spectrum utilization, increases network efficiency and extends network life. Consequently, this leads to the appearance of a new radio network paradigm for the IoT systems, called Cognitive Radio-enabled Low Power Wide Area Network (CR-LPWAN).

In CR-LPWANs for IoT systems, each device can sense the unused spectrum in both licensed and unlicensed spectrum bands. The unlicensed users can access the unutilized channels in licensed spectrum in the hope of improving the communication reliability and efficiency. That is, the licensed users can use the spectrum anytime, whereas unlicensed users can use the spectrum only when it is not used by the licensed user. More specifically, the cognitive radio-equipped unlicensed IoT device can select the most appropriate channel among the identified idle ones in the licensed spectrum band and can use it. If it is detected that a new licensed user arrives on the channel, the unlicensed IoT device moves to another spectrum hole and eventually vacates the channel. Hence, it becomes an important challenge for an unlicensed user to share licensed spectrum more efficiently without interfering with licensed users.

Our aim is to maximize the spectrum capacity for the unlicensed CR-enabled IoT network while ensuring that it never interferes with the licensed network. In this paper, therefore, we present a dynamic spectrum access strategy for cognitive radio-enabled LPWAN operating in both licensed bands and unlicensed bands. We also derive the blocking probability for the licensed users and carried traffic for the unlicensed users. In Section 2, related works are investigated and CR-LPWAN architecture is introduced in Section 3. A dynamic spectrum access model is presented and numerical analysis is given in Section 4 and Section 5, respectively. Results and discussions are presented in Section 6. Conclusions are given in Section 7.

## 2. Related Works

### 2.1. Low Power Wide Area Network (LPWAN)

Recently, the 3GPP (www.3gpp.org) has standardized the LTE-M (Long Term Evolution for Machines) and Narrowband IoT (NB-IoT) to provide cellular connectivity for Internet of Things (IoT) devices. These technologies are targeting a significant coverage improvement in order to support the devices located in deep indoor and rural areas [5,7]. However, the IoT devices may not rely solely on cellular connectivity, but on the radio access networks based on Low Power Wide Area Networks (LPWANs) in general (see Table 1). The most popular long-range LPWAN technologies are LoRa (www.lora-alliance.org), SigFox (www.sigfox.com) and Wi-SUN (www.wi-sun.org). While these technologies operate in sub-GHz unlicensed ISM bands and topologically adhere to a cellular-like structure, the associated operating principles are fundamentally different.

LoRa (Long Range) is a long-range, low-power, low-bitrate, wireless telecommunications system for the Internet of Things. In LoRa, data traffic can be initiated either by a sensor device or by an external entity for communicating with an actuator device [2,4,5]. A typical LoRa network has three different types of devices. The end-devices communicate to the gateway over a single wireless hop via LoRa. The gateway acts as transparent bridges between these end-devices and a central network server. LoRa is a good candidate for smart sensing technologies such as health monitoring, smart metering, and environment monitoring as well as industrial applications.

French company SigFox created an ultra-narrowband IoT communications system designed to support IoT deployments over long ranges. SigFox is a variation of the cellular system that enables remote IoT devices to connect to the access point [2,5]. SigFox claims that each access point can handle up to a million end-devices, with a coverage area of 30~50 km in rural areas and 3~10 km in urban areas. SigFox operates on the unlicensed spectrum of the 915 MHz frequency band in the U.S. and the 868 MHz frequency band in Europe, with the spectrum divided into 400 channels of 100 Hz.

Wi-SUN (wireless smart utility network) is a feasible solution to realize wide area IoT systems. Wi-SUN systems are based on IEEE 802.15.4g, which standardizes the physical layer specifications for outdoor low data rate wireless smart metering utility networks [6]. Wi-SUN has been applied to the smart metering systems for infrastructures such as electricity, gas and water. The system is composed of a high-performance base station (BS) and terminal devices with a sensor and meters.

### 2.2. Cognitive Radio for IoT

Cognitive radio is a promising technology for IoT. It can help overcome the problems that will arise due to the deployment of several objects connected to infrastructure through radio links. However, there are several issues that need to be addressed before cognitive radio technology can be used for IoT. There have been several studies on cognitive radio for IoT.

Wu et al. [22] present a comprehensive definition for cognitive IoT (CIoT), which is primarily inspired by the effectiveness of human cognition, and propose an operational framework of CIoT, which mainly characterizes the interactions among five fundamental cognitive tasks: perception action cycle, massive data analytics, semantic derivation and knowledge discovery, intelligent decision-making, and on-demand service provisioning.

Khan et al. [23] discuss how cognitive radio technology can be helpful for the IoT paradigm. This study mainly mentions that the future would be a world of trillions of IoT objects in need of continuous spectrum functionalities. Hence, traditional communication technologies would not withstand this situation and so, transformation from ordinary IoT objects to cognitive-coupled IoT objects would be inevitable to benefit from spectrum congestion situations. Thus, this study highlights CR functionalities, especially spectrum sensing in conjunction with cloud services to serve as self-reconfigurable IoT solutions for a number of applications.

Rawat et al. [24] survey novel approaches and recent advances on ongoing research directions related to cognitive radio in the context of Machine-to-Machine and Internet of Things. That is, this study reviews CR solutions that address generic problems of IoT including emerging challenges of autonomicity, scalability, energy efficiency, heterogeneity in terms of user equipment capabilities, complexity and environments, etc.

Nitti et al. [25] propose the utilization of the Social Internet of Things (SIoT) paradigm, according to which objects are capable of establishing social relationships in an autonomous way, with respect to the rules set by their owners. The resulting social network enables faster and trustworthy information/service discovery exploiting the social network of “friend” objects. The general approach is described according to which members of the SIoT collaborate to exchange channel status information.

Fadda et al. [26] propose a distributed spectrum sensing method based on an IoT architecture. The architecture comes in support of short-range video broadcasting for delivering commercial contents to several TV devices in an indoor scenario, involving several sensing-enabled objects in a joint manner. The main contribution of this study is the creation of new templates for the virtual objects (VOs) in order to represent the spectrum sensing capabilities of the involved devices. The VOs are combined at the higher levels of the IoT platform to find the relevant VOs and to decide whether the sensed channels can be used or not by secondary users.

### 2.3. Spectrum Access Strategies

Typically, the technique for unlicensed users to dynamically access the unutilized licensed bands in order to minimize unused spectral bands or white spaces is known as the dynamic spectrum access scheme. In this scheme, unlicensed users basically use unutilized licensed spectrum bands with no cost. When the licensed user starts to use the spectrum band, the unlicensed user has to make the band free and switch to another idle band. That is, the CR technique and dynamic spectrum access scheme are major solutions for increasing spectrum utilization in the cognitive radio sensor networks [15,27,28,29,30,31,32,33,34].

There have been several studies on spectrum access strategies in cognitive radio networks. Weiss et al. [8] discusses a new approach called spectrum pooling that enables public access to already licensed frequency bands. This notion basically represents the idea of merging spectral ranges from different spectrum owners (military, trunked radio, etc.) into a common pool. It represents the coexistence of two mobile radio systems within the same frequency range. The goal of spectrum pooling is to enhance spectral efficiency by overlapping a new mobile radio system on an existing one without requiring any changes to the actual licensed system.

Xing et al. [9] present the continuous-time Markov models of spectrum etiquette for dynamic spectrum access in open spectrum wireless networks. A random access protocol is proposed in order to show the achievement of airtime fairness. A distributed version of the channel access protocol that uses only local information is also proposed based on the homo egualis (HE) society model. These channel access protocols are extended to spectrum agile radios.

Zhu et al. [10] proposed a channel reservation scheme for cognitive radio spectrum handoff, which presents a general Markov chain analysis model for cognitive radio access in licensed bands. This scheme is similar to channel reservation used in a circuit-switched network, which allows the tradeoff between forced termination and blocking according to QoS (quality of service) requirements. That is, significant higher throughput can be achieved if a proper number of channels are reserved.

Etkin et al. [11] proposed a non-cooperative game for the distributed dynamic spectrum sharing, where all users are selfish and do not reveal their private information. Under this model, the authors study the spectrum sharing problem among multiple secondary users for interference-constrained wireless systems in a non-cooperative game framework. This study is focused on investigating self-enforcing spectrum sharing game rules and the corresponding game efficiency measured in total throughput obtained from available spectrum resources [12,13].

Byun et al. [27] proposed a new approach to solve a multi-objective problem using modified game theory while considering centralized spectrum allocations in resource-constrained wireless sensor networks. This scheme may also be feasible to allocate spectrum bands in a distributed manner by combining with a non-cooperative game algorithm. There are only a few studies that mention the deployment of cognitive radio in WSNs.

Jiang et al. [35] propose a dynamic spectrum access protocol for SUs (secondary users) confronted with the unknown behavior of PUs (primary users). In particular, this study focuses on the PUs’ interference caused by SUs’ dynamic access. This scheme analyzes the SUs’ dynamic behavior in the primary channel which is modeled as an ON-OFF process, where SUs’ behavior is proved to be a renewal process. This study finally aims at optimizing SUs’ performance under the constraints of the PU’s quality of service (QoS) and the SU network’s stability.

Jiang et al. [36] propose a method to jointly consider the spectrum sensing and access problem under two scenarios: a synchronous scenario where the primary network is slotted and a non-slotted asynchronous scenario. In order to model complex selfish SUs’ behaviors, the joint spectrum sensing and access problem are formulated as an evolutionary game and derive the evolutionarily stable strategy (ESS). Furthermore, this study has also designed a distributed learning algorithm for SUs to converge to the ESS, where each SU senses and accesses the primary channel with the probabilities learned purely from its own past utility history, and finally achieves the desired ESS.

Dudin et al. [37] propose a novel queueing model suitable for the optimization of access, in order to effectively solve the problems of the optimization of joint access of PUs and SUs. In this study, there are several types of PUs with different requirements for the service time and preemptive priority over SUs. SUs can share a server, while PUs occupy the whole server. The arrival flow is described by the marked Markovian arrival process. The service time distribution is of phase-type. The effect of retrials of SUs is taken into account.

Balapuwaduge et al. [38] propose two queueing schemes for SUs, which are based on the delay tolerance of interrupted elastic services. In multi-channel cognitive radio networks, SUs can achieve maximal capacity using dynamic channel assembling (CA) strategies. However, the channel allocation schemes suffer from high blocking and forced termination when primary users become active. In a multi-channel network with heterogeneous traffic, two queues are separately allocated to real-time and elastic users, and channel access opportunities are distributed between these two queues in a way that real-time services receive higher priority. Furthermore, continuous time Markov chain models are deployed to evaluate the performance of the proposed CA strategy with queues.

Jiang et al. [39] introduce several approaches for asynchronous spectrum sensing and asynchronous spectrum access, respectively, in a cognitive radio network, with which SUs can access the primary channels dynamically even without achieving synchronization with the PUs. This study discusses the key techniques, corresponding solutions, and potential application scenarios of the asynchronous spectrum sensing and access schemes. Specifically, this study presents two asynchronous spectrum sensing schemes under non-cooperative and cooperative scenarios, respectively.

Wang et al. [40] propose an analytical framework to quantify the queue dynamics of multi-SU multichannel cognitive radio networks (CRNs). The framework includes the important lower-layer mechanisms and settings, including spectrum sensing errors, medium-access control (MAC) protocols, link adaptation technologies such as adaptive modulation and coding (AMC) and automatic repeat request (ARQ), and limited buffer size. In order to analyze impacts on the quality-of-service (QoS) performance for SUs, the queue dynamics are modelled as a discrete-time finite-state Markov chain (FSMC). The performance metrics include average queueing delay, packet-loss rate, and effective throughput.

## 3. System Architecture

If IoT devices are equipped with cognitive radio capabilities in LPWANs, they can work as a CR-based IoT system. In a CR-enabled LPWAN, wireless IoT devices collaboratively convey their event readings over idle spectrum channels. Some of the LPWANs share a single channel for communication, and an event sensing trigger causes the IoT devices to generate the burst traffic packets. Hence, many IoT nodes, within the specific event area in the densely deployed CR-LPWAN, seek to seize the same channel simultaneously. This increases the call blocking probability or packet losses, which cause transmission delay and excessive power consumption. Hence, it is necessary for CR-LPWAN to access a proper channel opportunistically among multiple channels to mitigate this latent challenge.

### 3.1. Internet of Things (IoT) Systems

**Figure 1:**
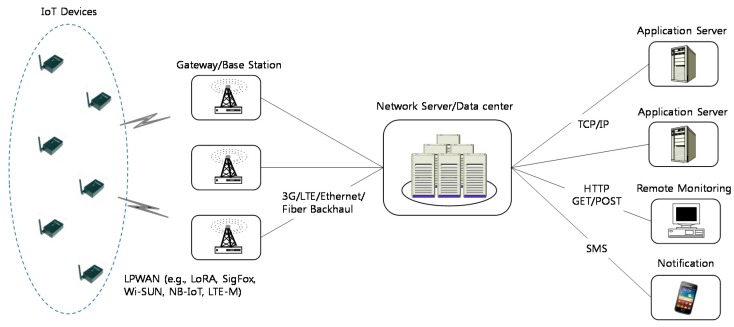
Low power wide area network (LPWAN) end-to-end network architecture for Internet of Things.

The IoT system has cellular-like backbone network architecture as shown in Figure 1. We assume that a CR-LPWAN is composed of IoT gateway (or base station) and IoT devices (or sensor nodes), which are uniformly distributed over the network area. These IoT devices can be assumed to adjust an arbitrary transmission power level for sustaining each data transmission. We also assume that there is at least one data transmission path from any IoT device to the gateway. The gateway node eventually collects all information acquired by each of the IoT devices. The collected information would finally be sent to the data server. In CR-LPWANs, each IoT device is responsible for determining its actions based on the local event readings. The local readings on each IoT sensor node need to be exchanged between the sensor nodes and central network server in order to broaden the sensing information on the entire network.

In addition to the event readings, IoT devices may exchange additional information with the network server. This information includes control data for spectrum allocation, and spectrum-handoff-aware channel determination, which depends on the specific topology. The typical duty cycle in CR-LPWANs, where IoT sensor devices are located geographically at random, depends on the sensing target. Path availability between the sensor nodes and the network server, and coverage intensity in CR-LPWANs can actually rely on the effective duty cycle.

### 3.2. Two Types of Radio Systems

**Figure 2:**
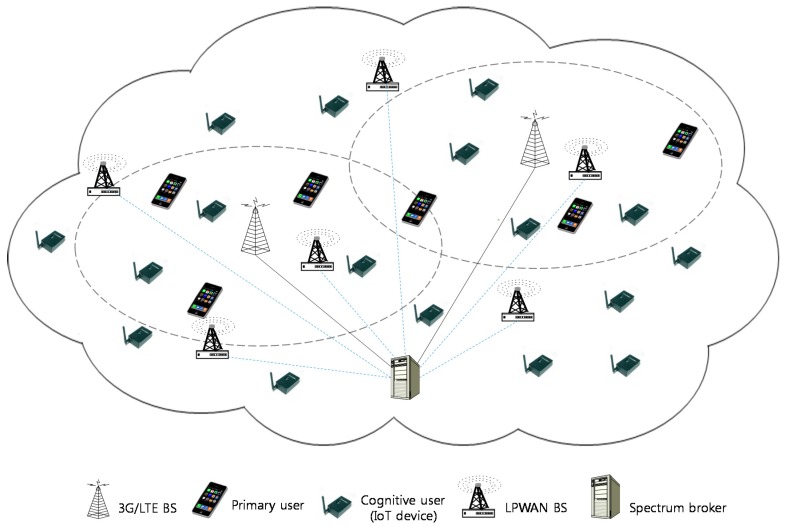
Two types of radio network architecture.

Primary radio networks (PRNs) and CR-enabled LPWANs are considered for the two types of radio systems as shown in Figure 2. The PRNs are mobile cellular networks where the fixed and licensed spectrum bands are often observed to be underutilized. CR-LPWANs work with PRNs and use their spectrum at a fixed frequency. Specifically, the spectrum channel contention between PRN and CR-LPWAN only takes place within the PRN radio spectrum range.

The licensed primary users (PUs) can exclusively use the spectrum bands allocated in PRNs. When PRN radio spectrum is not fully utilized, however, many spectrum holes in the PRN radio system can be temporarily used by the cognitive users (CUs) in CR-LPWANs. That is, CUs can identify and exploit these spectrum holes occurring in the PRN radio system. Specifically, CU should be able to exploit them to transmit in an opportunistic manner only when it does not interfere with any PUs.

In this architecture, IoT sensor devices send their reading values to the base station of LPWAN depending on spectrum availability by cognitive radio capability. The readings are eventually delivered to the data center, which is not indicated in Figure 2 for simplicity. That is, cognitive radio-equipped IoT devices communicate with each other according to cellular-like topology over both licensed PRN bands and unlicensed CR-LPWAN spectrum bands. This topology creates less control data in communication and makes more accurate results in spectrum sensing. If IoT devices in a CR-LPWAN have mobility, it produces a more dynamic topology and enlarges the existing challenges.

### 3.3. Spectrum Broker

In channel allocation for dynamic radio spectrum sharing, an accurate prediction of network performance is important. In this architecture, a central spectrum broker is responsible for allocating radio spectrum channels to cognitive unlicensed users as shown in Figure 2. A centralized sensing mechanism is performed for obtaining detailed information on unused frequencies although a decentralized sensing mechanism is also used. The transceiver on each IoT sensor device scans the spectrum availability and forwards the information of these spectrum holes to the spectrum broker via base station (or access point) in the respective network. The radio spectrum channel is allocated to the cognitive user on the IoT device depending on the number of unoccupied channels.

## 4. Dynamic Spectrum Access Model

The presence of spectrum holes in frequency spectrum allocation means that the radio spectrum is utilized inefficiently. Static radio spectrum access with the fixed resource allocation is not suitable for coping with spectrum shortages due to little resource sharing. On the contrary, dynamic spectrum access is good at handling spectrum holes or spectrum shortage by allocating the available bandwidth according to various approaches in an efficient manner. It should be able to apply different spectrum sharing to different systems as well as assign different fixed bandwidths to different systems.

Once media access control (MAC) or channel bandwidth allocation in LPWANs is well defined so that resource allocation can be easily made according to specific quality of service (QoS) in wireless applications, LPWANs can serve the traffic for wireless applications with strict QoS requirements. CR-LPWAN is especially efficient whenever the radio spectrum of a primary radio network (PRN) system with a large amount of resources becomes underutilized. Hence, in CR-LPWANs, the dynamic spectrum access is essential to efficient spectrum management for densely deployed IoT sensor nodes. CR-LPWAN is distinguished from traditional WSN by spectrum sensing functionality. Cognitive radio determines the vacant bands through the spectrum sensing capability by dynamically adjusting its operating parameters, and efficiently manages these available bands for improving the overall spectrum utilization.

### 4.1. Spectrum Access Model Under Two Types of Radio Systems

In this model, we consider the CR-enabled LPWAN overlapped with a primary radio network (PRN) such as the mobile cellular network. In this spectrum access model, frequency bands are used by two types of radio systems. One is the cellular network system operating in the licensed bands and the other is the wireless sensor network system operating in the unlicensed bands.

Similarly, there are two types of radio users, licensed PUs and unlicensed CUs. Typically, the unlicensed CU tries to use the available licensed channel. That is, the unlicensed users can access the licensed channel as long as they do not cause interference to the licensed users. Hence, when a PU begins to use an available licensed channel, the unlicensed CUs on IoT devices in CR-LPWAN, which operate on licensed bands in an opportunistic manner, must immediately detect the activity of licensed PU via spectrum sensing. That is, cognitive radio capability should be able to operate in licensed bands as well as unlicensed bands since the PUs do not always fully utilize the licensed spectrum bands.

**Figure 3:**
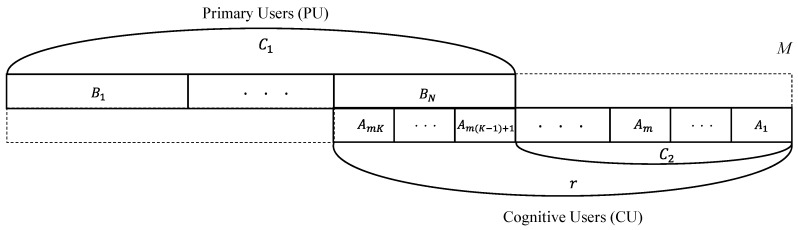
Spectrum band used by two types of radio systems.

Now let there be two types of radio users, PU and CU. In this model, the entire spectrum for two types of radio users actually consists of *M* sub-band units. Each primary user uses an A-band channel consisting of *m* sub-bands and each cognitive user uses a B-band channel consisting of only one sub-band. That is, one A-band channel is divided into *m* B-band channels. The A and B-band channels overlap with each other in the same part of the entire spectrum, as indicated in Figure 3, and the overlapped spectrum is used by both PUs and CUs. In more detail, a single cell of the primary radio network has *C*_1_ (= *N*) total channels, and CUs can only use some channels in the primary radio network beyond the channels (*C*_2_) allocated in a CR-LPWAN. The PUs have priority to use the overlapped spectrum and can reclaim any sub-bands used temporarily by CUs. Therefore, the presence of CUs is entirely transparent to the PUs.

In Figure 3, the primary users can use *N* channels and the cognitive users can use *r* (= *mK + C*_2_) channels where *K* = 0, 1, 2, …, *C*_1_. The process of spectrum occupation is modeled as a continuous time Markov chain. It is characterized by its states and transition rates. The *mC*_1_-(*M*-*r*) sub-band channels are shared by the primary users and cognitive users. In this case, the states are described by an integer pair (*i*, *j*), where *i* is the total number of primary users and *j* is the total number of the cognitive users. We assume the arrivals of primary users and cognitive users are both Poisson processes with arrival rates *λ*_1_ and *λ*_2_. The corresponding service times are exponentially distributed with rates *μ*_1_ and *μ*_2_.

### 4.2. Dynamic Spectrum Access Strategy

Typically, when the current channel condition becomes bad, the cognitive radio node can switch channel by spectrum handoff so that the on-going communication may be continued. In the proposed strategy, as the licensed PUs have the priority to use the allocated licensed spectrum bands over the unlicensed CUs in licensed spectrum bands, on-going CUs can be preempted by newly arriving PUs. That is, although the unlicensed users may temporally occupy the unused licensed sub-bands, they have to make these sub-bands vacant when the licensed users want to use them. Thus, in order for on-going CUs not to be dropped, the preempted CUs have to reconstruct the channels affected due to spectrum handoff. More specifically, when a PU begins to make a call for communication in licensed bands, the CUs have to move onto the vacant channel immediately after detecting the available channels in unlicensed bands by spectrum sensing. Whenever the cognitive users are newly preempted in primary licensed bands, however, spectrum handoff may succeed or fail depending on the number of channels occupied by cognitive users in licensed bands and the channels available in unlicensed bands.

In this strategy, CUs are allowed to overflow over the licensed spectrum allocated for PUs with the risk of being preempted by an arriving PU request. PUs can use up to *B_N_* channels from *B_1_* channel with preemptive priority over CUs in any PRN cell, and *A_1_* to *A_m(K_*_−*1)*_ channels are dedicated to CUs. If the CUs are preempted by an arriving PU, they have to immediately move onto the available unlicensed channels by spectrum handoff. If the preempted CUs fail to move onto the available channel due to there being no vacant channel, they can be queued at the head of the infinite cognitive user queue (CUQ) to wait for service instead of being cleared. The newly arriving CU call is also queued if there is no idle channel to accept a CU call. Whenever the unlicensed spectrum bands become available, the channel band is allocated to the CU request at the head of the queue. All the entries of CU requests entering this queue are served on a first come first served (FCFS) basis, and the size of queue is assumed to be infinite. Since CU call requests are not rejected in this strategy, the queueing delay may also be very large (nearly infinite) in the worst case. Although the queue size never becomes infinite in a practical situation, it is considered to be infinite in order to evaluate the maximum delay theoretically without any restrictions in queueing delay.

Upon arrival of PU calls, a PU call is blocked and cleared if the number of PU calls in service is equal to *B_N_* or there are not enough free channels. In this strategy, no priority is given to PU handoff call attempts over PU new call attempts for simplicity of the analysis model, so no difference exists between these call attempts; the blocking probability and the probability of hand-off attempt failure are the same.

In general, two extreme cases can be considered for these two types of radio systems. If the cutoff value *r* is restricted at *C*_2_ in Figure 3, the system will be two distinct spectrum band groups. However, this becomes clearly inefficient for IoT applications under LPWAN: CU calls might experience excessive delays while PU spectrum bands are unused. In another case, if the cutoff value *r* is set for CUs to fully access the licensed bands (*C*_1_ region) allocated for PUs, CUs can get more spectrum bands under heavy traffic of CU calls. However, random fluctuations in PU calls might severely harm CU call access. This situation also results in an excessive delay of CU calls due to the sudden preemption of many CU calls caused by arriving PU calls. This will be severe, since CU calls leave insufficient spectrum bands for accommodating an incoming PU call request, when the spectrum band ratio *m* (= PU/CU) for PU and CU calls is large. In other words, large bandwidth ratios for PU and CU calls may result in drastic spectrum inefficiency.

The system can be protected by restricting the maximum number (= *r*) of CU calls that can be in service at the same time. That is, the excessive delay of CU calls caused by the suddenly arriving PU calls can be protected by setting some restrictions on the CU calls. Therefore, it is natural to choose the cutoff values *r* that make the available channels for CU calls in licensed spectrum bands a multiple of the spectrum channel of a PU call. The cutoff value *r* needs to be adjusted in order to find good tradeoff between more spectrum availability and excessive delay experience for CU calls.

## 5. Numerical Analysis

Most analyses have been done with one type of traffic, which is usually assumed as a Poisson process. In this dynamic spectrum access model, heterogeneous types of traffics and their traffic characteristics are considered together. That is, the performance analysis for the admission control and the spectrum channel allocation should be based on heterogeneous traffic modeling. In particular, we need to consider the traffic characteristics which IoT sensor nodes generate in CR-LPWAN.

**Figure 4:**
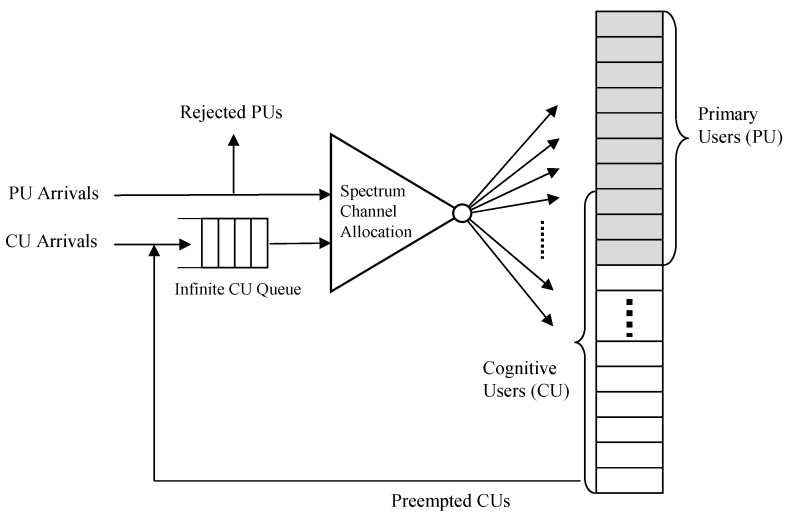
Dynamic spectrum access model for two types of radio systems.

Whenever an IoT sensor node wakes up from a sleep state, it can communicate with the outside world. In general, it depends on the applications for IoT devices in CR-LPWAN to decide when to turn off and on its radio transceiver. For simplicity, however, IoT sensor nodes are assumed to be turned on and off in a random style independent of other IoT devices with certain probability distribution. That is, IoT sensor nodes in CR-LPWAN are assumed to follow an independent random on/off schedule, where an individual IoT sensor node can be modeled as an alternating Markov renewal process with an on process and off process. Under the assumption that the channel requests of IoT sensor nodes will approach infinity, more specifically, the overall IoT sensor nodes can be modeled as Markov process, which can be derived as a special case of Markov renewal processes. Hence, the unlicensed spectrum access in CR-LPWAN can be modeled as a continuous time Markov chain. Without loss of generality, we can model the spectrum access with two types of radio systems illustrated in Figure 3 and Figure 4.

### 5.1. Analytic Model

We consider the wireless network system serving two types of radio calls for both primary user (PU) and secondary cognitive user (CU), respectively. The entire spectrum consists of *M* sub-band units. Each PU uses its own band consisting of *m* sub-bands (*m* < *M*) and each CU uses only one sub-band, and all sub-band units assigned to PU connection are occupied and released together. We assume that PU type (type 1) and CU type (type 2) calls arrive according to a Poisson process with mean arrival rate $\lambda_{k}$ for type *k* (*k* = 1, 2) calls, and that service time is exponentially distributed with a mean service time of $\mu_{k}{}^{-1}$ for type *k* calls. Then, the system can be modeled as a two-dimensional Markov process, characterized by$\;\{n_{1}\left(t\right),\;n_{2}\left(t\right)\}$ where $n_{1}\left(t\right)$ and $n_{2}\left(t\right)$ are the numbers of PU and CU calls in the system at time *t*, respectively, and the state space is represented by the set $\left\{s(n_{1},n_{2})|\;0\leq n_{1}\leq c_{1},\;n_{2}\;\geq 0\right\}$. Also, let the steady state probability that the system is in state $s(n_{1\;},n_{2})$ be $p(n_{1},n_{2})$. The steady state probability vector *p* (ordered lexicographically) is then partitioned as $p=\left(p_{0},p_{1},\cdots \right)$ where $p_{i}=\left(p\left(0,\;i\right),p\left(1,i\right),\cdots ,p\left(c_{1},i\right)\right)$, for i=0, 1, 2, ⋯. The vector *p* is the solution of equations pQ=0 and pe=1, where *e* and 0 are vectors of all ones and zeros, respectively. We can obtain the transition rate matrix *Q* of the Markov process, which is of the block-partitioned form for the proposed model and can be formulated as quasi-birth-and-death processes (QBDs). In order to make analyses of the model, matrix-geometric solutions can be applied, which are also described in Appendix A [41,42].

**Figure 5:**
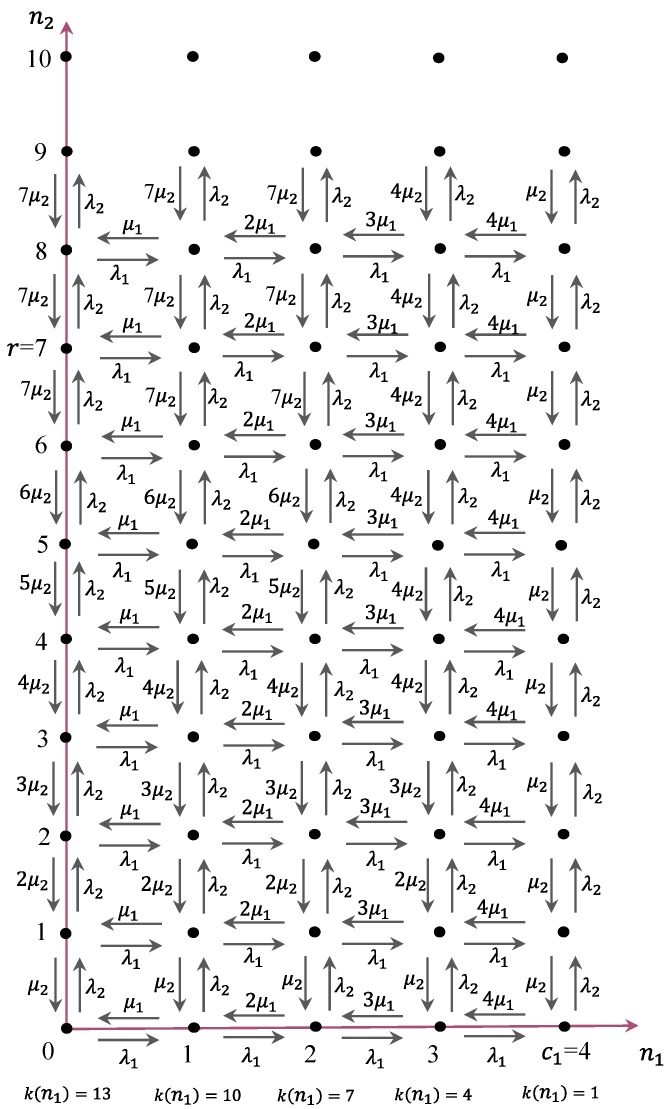
State transition diagram of proposed dynamic spectrum access strategy.

A state diagram of the system under the proposed strategy with *M* = 13, *m* = 3, *c*_1_ = 4, and *r* = 7 is shown in Figure 5, where k(n)=(M−n·m). From this figure, we can obtain the following transition rate matrix *Q* of the Markov process for the general system under the proposed strategy:

(1) $$Q=\left[\begin{array}{ccccccc} A_{0} & D & \; & \; & \; & \; & \;\\ B_{1} & A_{1} & D & \; & \; & \; & \;\\ \; & \cdot & \cdot & \cdot & \; & \; & \;\\ \; & \; & \cdot & \cdot & \cdot & \; & \;\\ \; & \; & B_{r-1} & A_{r-1} & D & \; & \;\\ \; & \; & \; & B_{r} & A_{r} & D & \;\\ \; & \; & \; & \; & \cdot & \cdot & \cdot \end{array}\right]$$

where the sub-matrices are defined for *i*, *j* = 0, 1, ⋯, *c*_1_ and *l* = 0, 1, ⋯, *r* (*m*·*c*_1_ + *r* ≥ *M*) by

(2) $${\;A}_{l}\left(i,j\right)=\{\begin{array}{c} \;\lambda_{1}\;\mathrm{if}\;i=j-1\\ \left(j+1\right)\mu_{1}\;\mathrm{if}\;i=j+1\\ {\;a}_{l}\left(i\right)\;\mathrm{if}\;i=j\;\\ \;0\;otherwise \end{array}$$

(3) $$B_{l}\left(i,j\right)=\{\begin{array}{c} \min\left(l,\;M-i\cdot m\right)\mu_{2}\;\mathrm{if}\;i=j\;\\ 0\;otherwise \end{array}$$

(4) $$D\left(i,j\right)=\{\begin{array}{c} \lambda_{2}\;\mathrm{if}\;i=j\;\\ \;0\;otherwise \end{array}$$

where *a_l_*(*i*) is the value that makes the sum of the row elements of *Q* to be equal to zero, and k(n)=(M−n·m).

To solve the equations pQ=0 and pe=1 with this transition rate matrix *Q*, we also apply Neut’s solution process [41] to the matrix *Q*. Let us first consider the stability of the system. The system is stable when the process *Q* is positive recurrent, i.e., $\pi B_{r}e>\pi De,$ where *π* is the stationary probability vector of $A_{r}+B_{r}+D=0$. Intuitively, the system is stable under the condition ($\lambda_{2}<r\mu_{2}$). Next, we determine the minimal non-negative matrix *R* of the matrix equation $R^{2}B_{r}+RA_{r}+D=0$ by iteration. Iterations can be made directly until $max_{\left(i,\;j\right)}\left[R_{i,j}\left(n+1\right)-R_{i,\;j}\left(n\right)\right]<\epsilon$ is satisfied, where *R*(*n*) is the *n*th iteration and ϵ is the degree of accuracy required. This matrix *R* gives the relationship $p_{k}=p_{k-1}R^{k-r+1},\;k\geq r.$ When the dimension of matrix *R* is large, considerable computational errors due to truncation and round-off may be accumulated in the iteration process. An effective method of $R^{2}B_{r}+RA_{r}+D=0$ is presented in Appendix A [41,42].

Next, the boundary probability vector $\tilde{p}=\left(p_{0},\cdots ,p_{c-1}\right)$ is the unique solution of $\tilde{p}T=0$ and $\tilde{p}e+p_{c_{1}-1}R{\left(I-R\right)}^{-1}e=1$, where the matrix *T* is a generator (*Te* = 0) given by

(5) $$T=\left[\begin{array}{ccccc} A_{0} & D & \; & \; & \;\\ B_{1} & A_{1} & D & \; & \;\\ \; & \cdot & \cdot & \cdot & \;\\ \; & \; & \cdot & \cdot & \cdot \\ \; & \; & B_{r-2} & A_{r-2} & D\\ \; & \; & \; & B_{r-1} & A_{r-1}+RB_{r} \end{array}\right]$$

The solution is then obtained by a two-step process: First, the rate matrix *R* is determined by iterative substitution in $R^{2}B_{r}+RA_{r}+D=0$. Second, once *R* is known, the *r*·*c*_1_ boundary probabilities $\tilde{p}$ are determined by solving a system of *r*·*c*_1_ linear equations. If *r* and *c*_1_ are large, the dimension of the matrix *T* for the boundary probabilities also becomes large. Therefore, it is difficult to provide the desired accuracy with the direct solution of the linear system $\tilde{p}T=0$ and $\tilde{p}e+p_{c_{1}-1}R{\left(I-R\right)}^{-1}e=1$. An alternative technique using decomposition can be applied to reduce the dimension. This procedure is also presented in Appendix A [41,42]. Once the matrix *R* and the boundary probability have been computed, the following performance measures are easily obtained.

### 5.2. Performance Measures

We consider a wireless network system serving two types of radio calls for both primary user (PU) and secondary. The mean system time *W*_1_ for PU calls only has the component of service time, i.e., *W*_1_ = 1/$\mu_{1}$. Since service quality for PUs is not a major concern here, the blocking probability for PU calls (or the forced termination probability) is simply

(6) $$P_{B_{1}}=\sum_{n_{2}=0}^{\infty }p\left(c_{1},n_{2}\right)=\sum_{n_{2}=0}^{r-1}p\left(c_{1},n_{2}\right)+{\left[P_{r-1}R{\left(I-R\right)}^{-1}\right]}_{\left[c_{1}\right]}$$

where []_[c1]_ denotes the *c*_1_th component of the vector in the bracket.

The mean number of CU calls in the system

(7) $$N_{2}=\sum_{n_{1}=0}^{c_{1}}\sum_{n_{2}=0}^{\infty }n_{2}p\left(n_{1},\;n_{2}\right)=\sum_{n_{1}=0}^{c_{1}}\sum_{n_{2}=0}^{r-1}n_{2}p\left(n_{1},\;n_{2}\right)+P_{r-1}R^{2}{\left(I-R\right)}^{-2}e+rP_{r-1}R{\left(I-R\right)}^{-1}e.$$

where the computational procedure on the second item with infinite parameter is also presented in Appendix A. Then, the mean dwell time for CU calls is from Little’s formula

(8) $$W_{2}=\frac{N_{2}}{\lambda_{2}}$$

Another important system performance is total carried traffic. For a given number of channels, a large carried traffic value implies efficient use of bandwidth. The carried traffic (*E*_1_) per PU in B-bands and the carried traffic (*E*_2_) per CU in both A-bands and B-bands can be easily calculated once the state probabilities are determined. Each measure is simply the average number of occupied channels per connection and can be given by

(9) $$E_{1}=\sum_{n_{1}=0}^{c_{1}}\sum_{n_{2}=0}^{\infty }n_{1}p\left(n_{1},\;n_{2}\right)=\sum_{n_{1}=0}^{c_{1}}\sum_{n_{2}=0}^{r-1}n_{1}p\left(n_{1},\;n_{2}\right)+\frac{1}{2}c_{1}\left(c_{1}+1\right)P_{r-1}R{\left(I-R\right)}^{-1}e.$$

and

(10) $$E_{2}=\sum_{n_{1}=0}^{c_{1}}\sum_{n_{2}=0}^{\min\left[k\left(n_{1}\right),r\right]}n_{2}p\left(n_{1},\;n_{2}\right)$$

Because a PU requires *m* sub-bands (B-band), the total carried traffic in a cell is given by

(11) E = mE1 + E2

To measure the combined service quality of PU and CU traffics, we also define the service quality factor of system as

(12) $$QT=\frac{W_{GT}(1-P_{B_{1}})}{\mu_{2}W_{2}}$$

where *W_GT_* is a weighted factor to adjust the difference between *P_B1_* and *W_2_*. Maximization of the quality factor means minimization of the blocking probability and system service delay.

## 6. Results and Discussions

**Figure 6:**
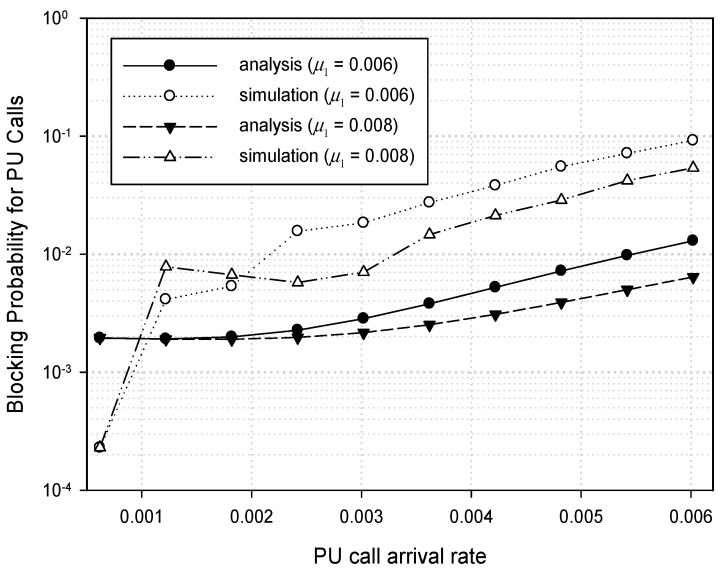
Blocking probability for PU calls.

In this dynamic spectrum access model are heterogeneous types of traffics and their traffic characteristics. In this section, the numerical analysis results are presented in order to illustrate the performance characteristics of a dynamic spectrum access strategy for a system with *M* = 13, *m* = 3, *c*_1_ = 4, *r* = 7 as shown in Figure 3. In Figure 6, we can see the blocking probability for PU calls when the PU traffic load *ρ*_1_ (= *λ*_1_/*μ*_1_) varies from *λ*_1_ = 0.0006 to 0.006 under *μ*_1_ = 0.006 and 0.008, respectively. Meanwhile, the CU traffic load *ρ*_2_ (= *λ*_2_/*μ*_2_) is kept constant at 0.01 with *λ*_2_ = 0.2 and *μ*_2_ = 20. From the dynamic spectrum access strategy shown in Figure 3, it can be seen that the blocking probability for PU calls only depends on *λ*_1_ and *μ*_1_ since the PU traffic is independent of CU traffic. Simulation is also performed together in order to verify the correctness of the mathematical analysis for the proposed dynamic spectrum access strategy. In Figure 6, the blocking probability for PU calls in the simulation is a little higher than that of the mathematical analysis overall. Before *λ*_1_ reaches 0.001, however, the blocking probability in the simulation is much lower than that of the mathematical analysis, and soon it becomes higher than the analysis result at around *λ*_1_ = 0.0012. In Figure 6, the difference between the simulation and the analysis of the blocking probability for PU calls is kept at a similar rate as *λ*_1_ increases to 0.006. Hence, the mathematical analysis can be verified with a similar upward tendency in distance. Figure 6 also shows that the blocking probability under *μ*_1_ = 0.006 is 13% more than under *μ*_1_ = 0.008 in both the simulation and the analysis as *λ*_1_ increases to 0.006.

**Figure 7:**
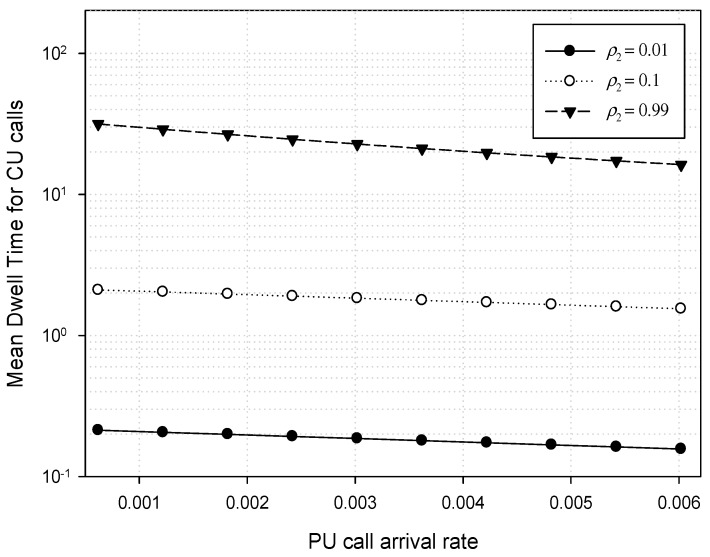
Mean dwell time for CU calls.

Figure 7 shows the mean dwell time for CU calls when the PU traffic load *ρ*_1_ varies from *λ*_1_ = 0.0006 to 0.006 with *μ*_1_ = 0.006. Meanwhile, the CU traffic load *ρ*_2_ is kept constant at 0.01, 0.1, and 0.99 (≈ 1.0) with *λ*_2_ = 0.2. Now we can consider the effect of the channel bandwidth ratio *m* on the mean dwell time of CU calls. In this example, CU call attempts can be overflowed into PU spectrum channels or can be queued in the CUQ when enough channels are not available. Under the heavy PU traffic, therefore, an arriving PU request makes *m* CU calls preempted, and then these CU calls can be queued unless they fail to move onto available channels. Consequently, the longer the time spent in the CU queue, the more the mean dwell time for CU calls increases. In Figure 7, however, we can see that the CU mean dwell time is slightly reduced as PU traffic load increases. This means that the licensed spectrum channels allocated for PUs are quickly occupied under heavy PU traffic, and hence no CU calls can overflow over the licensed spectrum.

Meanwhile, it is shown that the mean dwell time also increases when CU traffic load increases. Figure 7 shows the variation among the values in the mean dwell time according to different CU traffic load (*ρ*_2_) at 0.01, 0.1, and 0.99 (≈ 1.0). Since CU calls often fail to get channel allocations under the heavy CU traffic in this example, the CU calls can be queued more frequently. Now we can infer the effect of the ratio (*μ*_2_/*μ*_1_) of mean service time on the mean dwell time of CU calls. In Figure 7, the spectrum performance of CU calls is better when the service time ratio (*μ*_2_/*μ*_1_) is larger even though the PU service rate (*μ*_1_) is kept constant at 0.006. On the contrary, this means that the average dwell time of CU calls increases as the spectrum channel holding time of PU calls becomes longer. This phenomenon is common in the movable boundary strategy.

**Figure 8:**
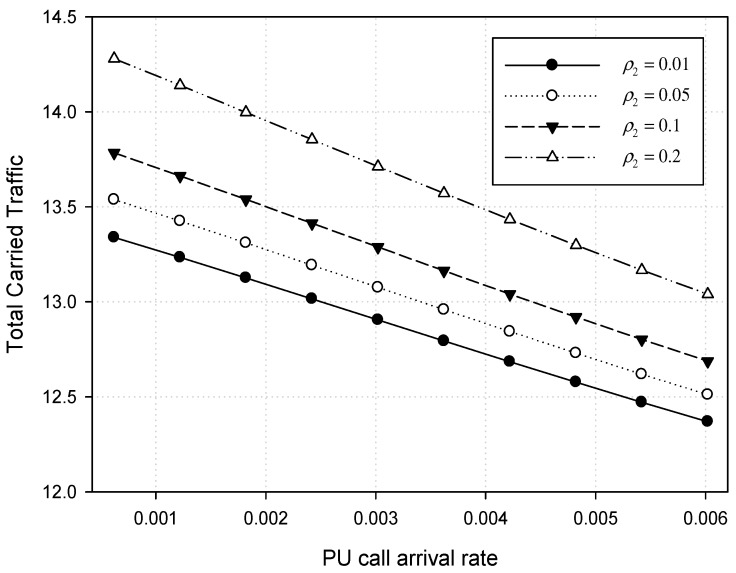
Total carried traffic for both PU and CU calls.

Figure 8 shows the total carried traffic when PU traffic load *ρ*_1_ varies from *λ*_1_ = 0.0006 to 0.006 under both *μ*_1_ = 0.006. Meanwhile, CU traffic load *ρ*_2_ is kept constant at 0.01, 0.05, 0.1, and 0.2 with *λ*_2_ = 0.2. Figure 8 also shows the variation among the total carried traffic according to different CU traffic load (*ρ*_2_) at 0.01, 0.05, 0.1, and 0.2. In this example, the total carried traffic also increases when CU traffic load increases. The CU channel allocation failure under the heavy CU traffic load often makes the CU calls queued more frequently. This may be explained as follows: if CU traffic enters into a few channels that the PU spectrum band leaves for the overloaded CU traffic, the continuous CU call attempts can make the queue grow. At this point, an arriving PU request makes *m* CU calls preempted as PU traffic increases, and this makes the CU queue size increase. In this situation, if the service time of the PU call becomes longer, this builds up a significant queue size. In Figure 8, however, we can see that the total carried traffic is slightly reduced as PU traffic load increases. This means that no CU calls can overflow over the licensed spectrum since the licensed spectrum channels allocated for PUs are quickly occupied under heavy PU traffic. That is, when the CU traffic load is switched to a normal situation, the queue size is continuously reduced and approaches its steady-state level. This phenomenon depends on the service time ratio, cutoff values, and the ratio of the spectrum bandwidth required for each type of traffic.

**Figure 9:**
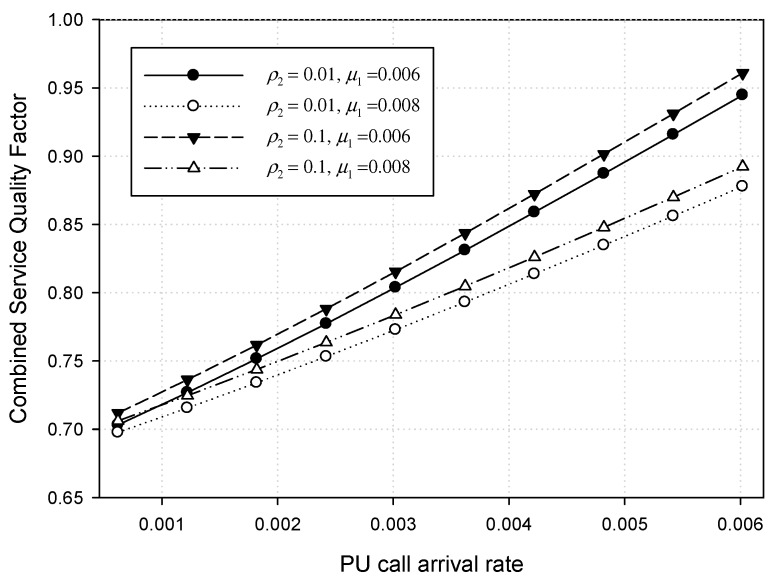
Combined service quality factor for both PU and CU calls.

Figure 9 shows the combined service quality factors when the PU traffic load *ρ*_1_ varies from *λ*_1_ = 0.0006 to 0.006 under *μ*_1_ = 0.006 and 0.008, respectively. Meanwhile, the CU traffic load *ρ*_2_ is kept constant at 0.01 and 0.1 with *λ*_2_ = 0.2 and weight factor *W_GT_* = 3.0. Figure 9 also shows that the combined service quality factor (CQF) increases at nearly the same rate as *λ*_1_ increases to 0.006. In this example, the CU arrival rate (λ_2_) is fixed as 0.2. That is, the lower the CU service rate (*μ*_2_) is, the higher the CU traffic load (*ρ*_2_) is. Hence, the higher the CU traffic load is, the better the CQF is. In Figure 9, it is shown that the CQF when *ρ*_2_ is 0.1 is better than the CQF when *ρ*_2_ is 0.01. Meanwhile, as shown in Figure 6, the higher the PU service rate *μ*_1_ is, the lower the blocking probability of the PU call requests is. That is, the higher the service rate (*μ*_1_) is, the better the CQF is. In Figure 9, however, we can see that the CQF when *μ*_1_ is 0.006 is better than the CQF when *μ*_1_ is 0.008. This can be explained as follows: the lower *μ*_1_ is, the longer the PU spectrum channel is occupied. In this situation, the rate that CU traffic overflows into the PU spectrum channel and then is preempted again, becomes lower than the situation with the higher *μ*_1_. This makes CU calls queued more frequently due to preemption and delayed more due to this queueing. Consequently, the CQF becomes lower in the higher service rate (*μ*_1_).

## 7. Conclusions

In this paper, we proposed a dynamic spectrum access strategy for Internet of Things (IoT) service operating in the licensed bands of mobile cellular networks as well as the unlicensed bands of the cognitive radio-enabled LPWANs (CR-LPWANs). Our aim is to maximize the spectrum capacity for the unlicensed CR-LPWAN while ensuring that it never interferes with the licensed mobile cellular network. The simulation and the numerical analysis for dynamic spectrum access strategy by using a matrix geometric approach are performed. Finally, we obtain the blocking probability of the licensed users, the mean dwell time of the unlicensed user, and the total carried traffic and the combined service quality for the licensed and unlicensed users. The results show that the proposed strategy can maximize the spectrum capacity for the unlicensed users on IoT devices. The total service quality is also good enough to support the applications for the unlicensed users on sensor (and actuator) based IoT devices. Moreover, the service quality of the licensed user is independent of the unlicensed users using a lot of different IoT applications.

## Figures and Tables

**Table 1:** Low Power Wide Area Radio Technologies for Internet of Things (IoT) Service.

	LoRa	SigFox	Wi-SUN	NB-IoT	LTE-M
Coverage	~11 km	~13 km	~5 km	~15 km	~11 km
Spectrum	800~900 MHz	800~900 MHz	900 MHz	700–900 MHz	700–900 MHz
Band	ISM/unlicensed	ISM/unlicensed	ISM/unlicensed	Cellular/licensed	Cellular/licensed
Bandwidth	125 KHz	0.1/0.6 KHz	0.1 KHz	200 KHz	20 MHz
Data Rate	~10 kbps	~100 bps	~300 kbps	150 kbps	~10 Mbps
TX Power	14 dBm	14/27 dBm	13/24 dBm	23/35 dBm	23 dBm
Roaming	N	N	N	Y	Y

## Appendix A

In the matrix-geometric solution, a quasi-birth-and-death process, called a QBD process, is a Markov process on the state space *E* = {(i,j), i ≥0, 1≤j≤m}, with infinitesimal generator $\tilde{Q}$, given by

(A1) $$\tilde{Q}=\left[\begin{array}{ccccccc} B_{0} & A_{0} & \; & \; & \; & \; & \;\\ B_{1} & A_{1} & A_{0} & \; & \; & \; & \;\\ \; & A_{2} & A_{1} & A_{0} & \; & \; & \;\\ \; & \; & A_{2} & A_{1} & A_{0} & \; & \;\\ \; & \; & \; & A_{2} & A_{1} & A_{0} & \;\\ \; & \; & \; & \; & A_{2} & A_{1} & \cdots \\ \; & \; & \; & \; & \; & \vdots & \ddots \end{array}\right]$$

where $B_{0}e+A_{0}e=B_{1}e+A_{1}e+A_{0}e=(A_{0}+A_{1}+A_{2})e=0$. The generator $\tilde{Q}$ is also assumed to be irreducible. The matrix $A=A_{0}+A_{1}+A_{2}$ is a finite generator.

**Theorem**: *The process* $\tilde{Q}$ *is positive recurrent if and only if the minimal nonnegative solution R to the matrix-quadratic equation*

(A2) $$R^{2}A_{2}+RA_{1}+A_{0}=0$$

*has all its eigenvalues inside the system and the finite system of equations*

(A3) $$\begin{array}{cc} x_{0}\left(B_{0}+RB_{1}\right) & =0\\ x_{0}{\left(I-R\right)}^{-1}e & =1 \end{array}$$

*has a (unique) positive solution* $x_{0}$.

Next, a computational procedure for the proposed dynamic spectrum access strategy is presented. In order to solve the minimal nonnegative matrix *R* of the matrix equation $R^{2}B_{r}+RA_{r}+D=0$, the following recursive iteration can be used directly:

(A4) $$R=-R^{2}B_{c_{1}}A_{c_{1}}^{-1}-DA_{c_{1}}^{-1}.$$

When the dimension of matrix *R* is large, considerable computational errors due to truncation and round-off may be accumulated in the iteration process. Some accuracy may be gained by rewriting the matrix equation as

(A5) $$R^{2}B_{c_{1}}+R(A_{c_{1}}-d\left(A_{c_{1}}\right))+D=-Rd\left(A_{c_{1}}\right)$$

where $d\left(A_{c_{1}}\right)$ is a diagonal matrix with its diagonal elements equal to the diagonal elements in $A_{c_{1}}$, respectively. Then the iteration equation becomes

(A6) $$R=-R^{2}B_{c_{1}}d\left(A_{c_{1}}^{-1}\right)-R\left[A_{c_{1}}d\left(A_{c_{1}}^{-1}\right)-I\right]-Dd\left(A_{c_{1}}^{-1}\right)$$

If the matrix $B_{c_{1}}$ is nonsingular (i.e., $m-nc_{2}\neq 0$), the following method can be also used, which converges more quickly than the above procedures. The matrix equation can be written as

(A7) $$R^{2}=-RA_{c_{1}}B_{c_{1}}^{-1}-DB_{c_{1}}^{-1}=RA+B$$

where $A=-A_{c_{1}}B_{c_{1}}^{-1}$, $B=-DB_{c_{1}}^{-1}$. The *ij* component of (A7) is given by

(A8) $$\sum_{k=0}^{c_{2}}r_{ik}r_{kj}=\sum_{k=0}^{c_{2}}r_{ik}a_{kj}+b_{ij}$$

Then, this equation is used as an iteration procedure for $r_{ij}$. Thus, for diagonal elements $r_{ii}$, the following expression is obtained:

(A9) $$r_{ii}^{2}-r_{ii}a_{ii}+\sum_{k=0,k\neq i}^{c_{2}}r_{ik}\left(r_{ki}-a_{ki}\right)-b_{ii}=0$$

Which yields the solution

(A10) $$r_{ii}=\frac{1}{2}\left[a_{ii}-{\left[a_{ii}^{2}-4\left(\sum_{k=0,k\neq j}^{c_{2}}r_{ik}\left(r_{ki}-a_{ki}\right)-b_{ii}\right)\right]}^{\frac{1}{2}}\right]$$

When a negative root is taken to obtain eigenvalues less than 1, for the off-diagonal elements, the corresponding equations are $\left(r_{ii}+r_{jj}-a_{jj}\right)r_{ij}+\sum_{k=0,k\neq i,j}^{c_{2}}r_{ik}r_{kj}-\sum_{k=0,k\neq j}^{c_{2}}r_{ik}a_{kj}-b_{ij}=0$ and

(A11) $$r_{ij}=\frac{\left[\sum_{k=0,k\neq j}^{c_{2}}r_{ik}a_{kj}+b_{ij}-\sum_{k=0,k\neq i,j}^{c_{2}}r_{ik}r_{kj}\right]}{\left(r_{ii}+r_{jj}-a_{jj}\right)}$$

For the obtained *R*, the accuracy can be checked from the equality

(A12) $$RB_{c_{1}}e-De=0$$

If $c_{1}$ and $c_{2}$ are large, the dimension of the matrix *T* for the boundary probabilities also becomes large. Consequently, it is difficult to have the desired accuracy in a direct solution of the linear system. An alternative technique that utilizes decomposition can be applied to reduce the dimension of the problem. First, $\tilde{p}T=0$ and $\tilde{p}e+p_{c_{1}-1}R{\left(I-R\right)}^{-1}e=1$ in the expression (5) are rewritten as

(A13) $$p_{0}A_{0}+p_{1}B_{1}=0$$

(A14) $$p_{k-1}D+p_{k}A_{k}+p_{k+1}B_{k+1}=0\;for\;k=1,\;\cdots ,\;c_{1}-2$$

(A15) $$p_{c_{1}-2}D+p_{c_{1}-1}(A_{c_{1}-1}+RB_{c_{1}})=0$$

The dimension of the sub-matrix in the above equation is $\left(c_{2}+1)\times (c_{2}+1\right)$. Now we define the matrix $H_{k},\;k=1,\;\cdots ,\;c_{1}-2$ as

(A16) $$H_{k}=\left[\begin{array}{cc} -A_{k}D^{-1} & I\\ -B_{k+1}D^{-1} & 0 \end{array}\right]$$

From (A13), the following expressions are obtained:

(A17) $$\left(p_{k-1},p_{k}\right)=\left(p_{k},p_{k+1}\right)H_{k}$$

and

(A18) $$\left(p_{0},p_{1}\right)=\left(p_{c_{1}-2},p_{c_{1}-1}\right)H_{c_{1}-2}H_{c_{1}-3}\ldots H_{1}$$

By combining (A13), (A15), and (A18), the following expression is also obtained:

(A19) $$p_{c_{1}-1}\left(-\left[A_{c_{1}-1}+RB_{c_{1}}\right]D^{-1},I\right)H_{c_{1}-2}\ldots H_{1}\left[\begin{array}{c} A_{0}\\ B_{1} \end{array}\right]=0$$

Consequently, using (A17), (A19) and the normalization condition ($\tilde{p}T=0$ and $\tilde{p}e+p_{c_{1}-1}R{\left(I-R\right)}^{-1}e=1$), the steady-state probabilities can be determined.

Finally, the computational procedure is presented for getting the mean number of CU calls in the dynamic spectrum access strategy. $N_{2}$ is given as following:

(A20) $$N_{2}=\sum_{n_{1}=0}^{c_{1}}\sum_{n_{2}=0}^{\infty }n_{2}p\left(n_{1},\;n_{2}\right)=\sum_{n_{1}=0}^{c_{1}}\sum_{n_{2}=0}^{r-1}n_{2}p\left(n_{1},\;n_{2}\right)+\sum_{n_{1}=0}^{c_{1}}\sum_{n_{2}=r}^{\infty }n_{2}p\left(n_{1},\;n_{2}\right)$$

Here, the second item with infinite parameter can be solved as following:

(A21) $$\begin{array}{c} \sum_{n_{1}=0}^{c_{1}}\sum_{n_{2}=r}^{\infty }n_{2}p\left(n_{1},\;n_{2}\right)\;\left(P_{k}=P_{r-1}R^{k-r+1},\;k\geq r\right)\\ =\sum_{n_{1}=0}^{c_{1}}\sum_{n_{2}=r}^{\infty }n_{2}P_{n_{2}}=\sum_{n_{1}=0}^{c_{1}}\sum_{n_{2}=r}^{\infty }n_{2}P_{r-1}R^{n_{2}-r+1}\;\\ =\sum_{n_{1}=0}^{c_{1}}P_{r-1}R^{-\left(r-1\right)}\sum_{n_{2}=r}^{\infty }n_{2}R^{n_{2}}\;=\sum_{n_{1}=0}^{c_{1}}P_{r-1}R^{-\left(r-1\right)}R\frac{\partial }{\partial R}\left(\frac{R^{r}}{I-R}\right)\\ =\sum_{n_{1}=0}^{c_{1}}P_{r-1}R^{-\left(r-1\right)}R\left(\frac{rR^{r-1}\left(I-R\right)+R^{r}}{{\left(I-R\right)}^{2}}\right)\\ =\sum_{n_{1}=0}^{c_{1}}P_{r-1}\left(\frac{rR\left(I-R\right)+R^{2}}{{\left(I-R\right)}^{2}}\right)\\ =\;\sum_{n_{1}=0}^{c_{1}}\left[rP_{r-1}R{\left(I-R\right)}^{-1}+P_{r-1}R^{2}{\left(I-R\right)}^{-2}\right]\;\\ =P_{r-1}R^{2}{\left(I-R\right)}^{-2}e+rP_{r-1}R{\left(I-R\right)}^{-1}e \end{array}$$
